# Exploring the Potential of Grape Pomace Extract to Inhibit Thermo-Oxidative Degradation of Sunflower Oil: From Routine Tests to ATR-FTIR Spectroscopy

**DOI:** 10.3390/foods11223674

**Published:** 2022-11-16

**Authors:** Mariana-Atena Poiana, Diana Moigradean, Delia-Gabriela Dumbrava, Isidora Radulov, Diana Nicoleta Raba, Adrian Rivis

**Affiliations:** 1 Faculty of Food Engineering, University of Life Sciences “King Michael I” from Timisoara, Calea Aradului 119, RO-300645 Timisoara, Romania; 2 Faculty of Agriculture, University of Life Sciences “King Michael I” from Timisoara, Calea Aradului 119, RO-300645 Timisoara, Romania; 3 Faculty of Management and Rural Tourism, University of Life Sciences “King Michael I” from Timisoara, Calea Aradului 119, RO-300645 Timisoara, Romania

**Keywords:** grape pomace extract, convective heat exposure of sunflower oil, thermo-oxidative degradation, chemical indices, ATR-FTIR spectroscopy, inhibitory effect

## Abstract

Exploring new sources of natural antioxidants is of great interest to edible oil producers, in line with the toxicological problems generated by the use of synthetic antioxidants. This study assesses the potential of lyophilized Pinot Noir grape pomace extract (GPE) to enhance the sunflower oil stability against thermo-oxidative damage compared to BHT during a prolonged exposure to convective heat at 185 °C. Oil thermo-oxidation was monitored based on specific indices such as peroxide value (PV), *para*-anisidine value (*p*-AV), inhibition of oil oxidation (IO), total oxidation (TOTOX) value, conjugated dienes and trienes (CDs, CTs), but also by attenuated total reflectance Fourier transform infrared spectroscopy (ATR-FTIR), where absorbance ratios A 3009 cm^−1^/A 2922 cm^−1^ (RI), A 3009 cm^−1^/A 2853 cm^−1^ (RII), A 3009 cm^−1^/A 1744 cm^−1^ (RIII) and RIV = A 1744 cm^−1^/A 2922 cm^−1^ (RIV) were investigated. GPE showed a significant inhibitory effect on oil thermo-oxidation and this response was concentration-dependent. Substantial decreases in the investigated indices, compared to the control without added antioxidants, were obtained after 4 h and 8 h of heat exposure of the 800 ppm GPE sample: PV (47%; 42%), *p*-AV (38%; 33%), IO (54%; 46%), TOTOX (41%; 37%), CDs (46%; 39%), CTs (44%; 29%). Oil exposure to heat resulted in changes in RI–RIV attributed to the reduction in the degree of unsaturation, in response to primary and secondary lipid oxidation. FTIR spectroscopy can be used to differentiate untreated and heat-treated oils based on the absorbance ratios. An inhibitory effect close to that of BHT was achieved by 500 ppm GPE, while a dose of 800 ppm provided greater protection against thermo-oxidation. Our results promote GPE as a natural additive to limit the thermo-oxidative damage of plant oils.

## 1. Introduction

The long-term use of edible plant oils is widely practiced in both domestic and industrial food thermal applications. Often, no attention is paid to the duration of the heat treatment or even to the reuse of thermally degraded oil. The exposure of vegetable oils to thermal stress, of which is associated with high temperatures, affects the structure of fatty acid in triglyceride molecules, resulting in alterations of the physico-chemical properties of vegetable oils due to the changes in chain length, degree of unsaturation and the position of unsaturation [1]. The heat treatment of culinary oils is responsible for complex chemical reactions leading to lipid peroxidation [2].

The quality and nutritional properties of both cooking oils and fried food products are affected by the generation of a large number of volatile or non-volatile compounds, such as free fatty acids, alcohols, aldehydes and ketones, cyclic and epoxy compounds, hydrocarbons and trans isomers [3,4].

Edible oils consist of a high percentage of polyunsaturated fatty acids, which makes them susceptible to oxidative damage during food applications which involve high temperatures. Currently, the exploration of new sources of natural antioxidants is a great challenge for edible oil producers and the food industry, in line with the safety aspects associated with the use of synthetic antioxidants [5,6].

Grape pomace, recognized due to its relevant natural dietary antioxidants content, is an underutilized by-product generated in huge quantities in the wine industry, leading to a waste-management problem [7,8]. Among bioactive compounds, polyphenols, such as anthocyanins, flavanols, catechins, and proanthocyanidins, play an important role due to their health-promoting potential [9,10]. Grape pomace valorization by the extraction of phenolic compounds opens new trends toward the wide use of the obtained extracts in food purposes [11,12,13]. Grape marc extracts are potential sources of natural antioxidants [14,15,16] that may be considered for the oil industry to replace or decrease the dose of synthetic antioxidants. Their use as natural antioxidants may provide an effective strategy to enhance the oxidative stability of vegetable oils during a prolonged exposure to high temperatures. The use for this purpose depends largely not only on their chemical composition, but also on their ability to limit the oxidative degradation in heat-treated oils.

Although the results reported so far recommend the use of grape pomace extracts as natural food antioxidants [11,12], there are still no systematic studies on their potential to inhibit lipid oxidation in edible oils subjected to thermal stress. The studies conducted in this regard have referred to the use of extracts obtained from different parts of marc, seeds and peels, and have referred less to the extracts from the whole grape pomace [17]. The potential of grape seed extract to inhibit or delay lipid oxidation has been demonstrated in various food applications, such as meat preservation [18,19], the storage of extruded corn chips [20], soybean oil [21] and the high-temperature heating of sunflower oil [22]. Additionally, red grape seeds and peel extract were able to stabilize sunflower oil [23].

Different methods are widely used as routine tests to evaluate the oxidative quality parameters of vegetable oils. The most common are used to quantify the concentration of primary and secondary oxidation products. The peroxide value (PV) is an indicator for the concentration of hydroperoxides as primary oxidation products, while the *para*-anisidine value (*p*-AV) provides a measure of the level of secondary oxidation products, such as aldehydes, carbonyls, trienes and ketones, generated under oxidative conditions [24,25]. After the formation of hydroperoxides, as oxidation progresses, the unconjugated double bonds of the natural unsaturated lipids are rearranged, leading to conjugated dienes, which exhibit an intense absorption at 232 nm. When polyunsaturated fatty acids containing three or more double bonds undergo oxidation processes, the conjugation can be extended to include another double bond. As a result, conjugated trienes, with typical absorption at a wavelength of 268 nm, are formed. In addition to PV and *p*-AV, the variations in the specific extinctions of UV (K232 and K268) are used to assess the extent of the lipid oxidation process [26].

These methods have some shortcomings as they require time, and reagents and are not able to offer information on the real chemical composition of the generated products, and therefore, provide only a limited insight into oxidative damage. Therefore, understanding changes that occur at a high temperature is of particular interest.

In this regard, attenuated total reflectance–Fourier transform infrared (ATR-FTIR) spectroscopy, one of the most well-known spectroscopic techniques which works well in the middle infrared region, has been applied for the characterization of the oxidation degree of heat-treated edible oils [27,28,29]. FTIR spectroscopy has been successfully used in recent years for the rapid monitoring of the oxidative status of heat-treated vegetable oils based on the vibrations of different chemical groups that occur at specific wavelengths in the infrared spectrum. Several absorbance changes have been found between non-oxidized and oxidized vegetable oils as a result of thermal oxidation during high-temperature exposure. Thus, the main changes in the infrared spectra bands can be interpreted in relation to the lipid oxidation mechanism [25,27,30]. Additionally, the use of some ratios of the absorbance values of specific bands recorded in the FTIR spectra instead of absolute absorbance values for assessing the changes during the oxidative processes has been studied [30,31,32]. This technique combines rapidity and sensitivity with minimal sample preparation and a low consumption of solvents and chemical reagents [27]. Moreover, FTIR spectroscopy can be included in the range of environmentally friendly techniques that support the green analytical chemistry.

In line with safety concerns arising from the use of synthetic additives and to satisfy the consumer’s requirements and preference for natural foods, this research was planned to explore the effectiveness of freeze-dried grape pomace extract to inhibit thermo-oxidation in sunflower oil during a prolonged exposure to heat at 185 °C in a convention oven for up to 8 h, using both classical chemical indices and ATR-FTIR spectroscopy. This study represents one of the few approaches to evaluate the potential of grape pomace extract to limit the thermo-oxidative damage of edible plant oils exposed to high temperatures.

## 2. Materials and Methods

### 2.1. Processing of Grape Pomace Extract (GPE)

Raw grape pomace (RGP), a main by-product in the winemaking of Pinot Noir grape variety, has been taken from western Romania (Recas Winery, Timis County). RGP was dried at a moderate temperature of 60 °C for 8 h daily for three consecutive days, in a Binder drying oven (Binder GmbH, Tuttlingen, Germany); the obtained dried grape pomace (DGP) was ground with a Grindomix GM 200 cutting mill (Retsch GmbH, Haan, Germany). The ground material was sifted with a 60-mesh sieve and further used for the extraction of bioactive compounds in agreement with previous studies on this topic [10,33,34], as follows: 10 g of ground sample was treated with 200 mL of 70% (*v/v*) ethanol and placed for 48 h in a Promax 1020 shaker (Heidolph Instruments GmbH & Co. KG, Schwabach, Germany) at 25 °C. The mixture was vacuum filtered, and the clear fraction was centrifuged at 4500 rpm for 10 min. The supernatant was vacuum-concentrated at 55 °C with a Heidolph Laborota 4000 rotary evaporator (Heidolph Instruments GmbH & Co. KG, Schwabach, Germany). The concentrated extract was lyophilized in a laboratory freeze dryer TFD5503 (Ilshin Lab Co., Ltd., Gyeonggi-do, Seoul, Republic of Korea). The freeze-dried grape pomace extract (GPE) was stored in a freezer at –18 °C.

### 2.2. Sunflower Oil Sample Preparation and Heating Protocol

Refined sunflower oil (SFO), with no added synthetic antioxidants, was used in this study. GPE was added to the SFO samples at three concentrations—200 ppm, 500 ppm and 800 ppm (*w/v*). The oil samples with added GPE as a natural additive were noted as SFO + 200 ppm GPE, SFO + 500 ppm GPE, and SFO + 800 ppm GPE. A sample of SFO was also prepared with the synthetic antioxidant butylated hydroxytoluene (BHT), which was added in a dose of 200 ppm, corresponding to the legal limit (SFO + 200 ppm BHT). As a control, a sample of SFO without any antioxidant was used.

To ensure antioxidant dispersion in the oil, both GPE and BHT were separately mixed for 10 min before application, with a minimum amount of ethyl alcohol 96% (*v/v*) in an ultrasonic water bath. Then, the mixtures were added to SFO, and the prepared oil samples were subjected to vacuum evaporation [22,35]. The same amount of ethanol was used to dissolve BHT, and GPE was added to prepare the control.

The heat treatment of oil samples was carried out as follows: 20 ± 0.5 g of SFO weighed in Pyrex Petri dishes (10 cm inner diameter) was placed without lids in the convection oven AC 60 Froilabo of 1000 W (Froilabo, Meyzieu, France) and continuously heated at 185 ± 3 °C for two periods, 4 h and 8 h, respectively. During the SFO sample’s exposure to heat, the oil temperature was measured with a calibrated chromel-alumel thermocouple HI 935009 (Hanna Instruments Inc, Woonsocket, RI, USA). After each heating period, the samples were removed from the oven, cooled to room temperature, poured into brown glass bottles and placed under refrigeration conditions (4–5 °C) until the analysis. The oil samples were kept at room temperature (25 °C) for 24 h prior to analysis.

### 2.3. Analysis of Raw Grape Pomace, Dried Grape Pomace and Grape Pomace Extract

#### 2.3.1. Moisture Content 

The moisture content of RGP, DGP and GPE was evaluated by the official method of the AOAC [36].

#### 2.3.2. Assessment of Total Antioxidant Capacity by the Ferric-Reducing Antioxidant Power Assay

The antioxidant capacity of RGP, DGP and GPE was evaluated comparatively with standard substances AA and BHT by the ferric-reducing antioxidant power (FRAP) assay proposed by Benzie and Strain [37]. The assay is based on the potential of antioxidant compounds in the investigated samples to reduce Fe^3+^ from the Fe^3+^-2,4,6-tris(2-pyridyl)-1,3,5-triazine (TPTZ) complex to the Fe^2+^ form at 37 °C and pH 3.6 in a sodium acetate buffer solution. The reduction process results in changes in the absorbance value measured at 595 nm. To prepare the ethanolic extracts of RGP and DGP, 2 g of ground samples was treated with 20 mL of 70% (*v/v*) ethanol for 2 h at 25 °C, under continuous stirring using a Promax 1020 shaker (Heidolph Instruments GmbH & Co. KG, Schwabach, Germany). After extraction, the samples were filtered using Whatman filter paper No 1. Before analysis, the clear ethanolic extracts were diluted 1:100 (*v/v*) with distilled water. Regarding GPE, BHT and AA, 0.1 g of each sample was mixed with 10 mL of 70% (*v/v*) ethanol for 10 min, and then was filtered; before the analysis, the obtained filtrate was diluted 1:1000 (*v/v*) with distilled water. Next, a 0.5 mL aliquot of diluted extracts was added to 2.5 mL of FRAP reagent, and the samples were incubated at 37 °C for 30 min before measuring the absorbance at 595 nm against a blank sample prepared in the same conditions. A calibration curve using standard solutions of Fe^2+^ with concentrations ranging from 0.05 to 0.4 μM Fe^2+^/mL was prepared. The absorbance at the specified wavelength was read with a Specord 205 UV–VIS spectrophotometer (Analytik Jena Inc., Jena, Germany). The FRAP value of the sample was reported as µM Fe^2+^ equivalents per g of dry substance (d.s.).

#### 2.3.3. Assessment of Radical Scavenging Activity by 1,1-Diphenyl-2-picrylhydrazyl (DPPH) Assay

The radical scavenging activity of RGP, DGP and GPE compared to standard substances AA and BHT was determined by the 1,1-diphenyl-2-picrylhydrazyl (DPPH) assay [38]. To prepare the ethanolic extracts of RGP and DGP, a quantity of each ground sample that provided 1 g of dry substance (d.s.) was subjected to extraction with 10 mL of 70% (*v/v*) ethanol for 2 h at 25 °C under continuous stirring, followed by filtration with Whatman filter paper No 1. The clear filtrates were further diluted with 70% (*v/v*) ethanol to obtain a final concentration in the assay mixture of 200, 300, 400, 500 and 600 μg d.s./mL. For GPE, AA and BHT, solutions with concentrations of 10, 20, 30, 40 and 50 μg/mL in 70% (*v/v*) ethanol were also prepared. A 1 mL aliquot of the solutions prepared in the above-mentioned concentrations was mixed with 2.5 mL of a 0.1 mM solution of 1,1-diphenyl-2-picrylhydrazyl (DPPH) in 70% (*v/v*) ethanol. The mixtures were vortexed and incubated for 30 min at room temperature in the dark, then the absorbance was read at 517 nm against a sample consisting of 70% (*v/v*) ethanol. A control sample was prepared as shown above from a 2.5 mL solution of 0.1 mM DPPH in 70% (*v/v*) ethanol and 1 mL of 70% (*v/v*) ethanol. The percent inhibition of DPPH was obtained based on the relationship shown in Equation (1):

(1) $$\mathrm{DPPH}\;\mathrm{inhibition}\;(\%)=\frac{A_{c}-A_{s}}{A_{c}}\times 100$$

where Ac is the absorbance of the control, and As is the absorbance in the presence of the sample. 

Radical scavenging activity was expressed as a half-maximal inhibitory concentration (IC50), meaning the sample concentration required to decrease the initial concentration of free DPPH radical by 50% under particular experimental conditions. IC50 was calculated from a linear regression curve of DPPH inhibition (%) versus sample concentrations.

#### 2.3.4. Evaluation of Total Phenolic Content (TPC)

The total phenolic content (TPC) of RGP, DGP and GPE was assessed using the Folin–Ciocalteu colorimetric method [39]. Briefly, a 0.5 mL aliquot of the diluted solution of GPE prepared in the case of the FRAP test was mixed with 2.5 mL of the Folin–Ciocalteu reagent (Sigma-Aldrich Chemie GmbH, Munich, Germany), and was previously diluted 1:10 (*v/v*) with distilled water. After 5 min, 2 mL of the 7.5% (*w/v*) Na_2_CO_3_ aqueous solution was added and the mixture was placed in the dark for 2 h at 25 °C. Then, the absorbance was measured at 750 nm versus a blank sample, which was prepared in the same experimental conditions. The calibration curve was plotted using standard gallic acid solutions (Fluka, Madrid, Spain) with concentrations ranging from 10 to 100 mg GAE/L. TPC was reported as mg gallic acid equivalents (GAE) per gram of dry substance (d.s.).

### 2.4. Routine Tests to Evaluate the Progress of Sunflower Oil Thermo-Oxidation 

The magnitude of lipid oxidation was evaluated by measuring specific chemical indices used as routine tests such as peroxide value (PV), *para*-anisidine value (*p*-AV), conjugated dienes and conjugated trienes. Additionally, the inhibitions of oil oxidation (IO) and total oxidation (TOTOX) were calculated. 

PV provides a quantitative measure of hydroperoxide levels and was iodometrically evaluated according to the ISO standard methods (3960:2009) [40]. The inhibition of oil oxidation was calculated by the relationship provided in Equation (2) [35]:

(2) $$\mathrm{IO}\;(\%)=(1-\frac{\mathrm{increase}\;\mathrm{in}\;\mathrm{PV}\;\mathrm{of}\;\mathrm{oil}\;\mathrm{sample}}{\mathrm{increase}\;\mathrm{in}\;\mathrm{PV}\;\mathrm{of}\;\mathrm{control}})\times 100$$

The *para*-anisidine value (*p*-AV) provides a measurement of aldehydic compounds formed by secondary lipid oxidation. This index was determined according to the ISO standard method (6885:2008) [41]. This method is based on the reactivity of the aldehyde carbonyl bond on the amine group of *para*-anisidine, which results in the formation of a Schiff base exhibiting the maximum absorption at 350 nm. A total of 2 g of SFO (w) was dissolved in 25 mL of isooctane and the absorbance (AI) was measured at 350 nm against an isooctane blank. Then, 5 mL of this solution was transferred into a test tube, where 1 mL of the anisidine solution (0.25% in glacial acetic acid) was added. After 10 min, the absorbance (A_II_) was measured at 350 nm against a sample prepared from 5 mL of isooctane and 1 mL of the anisidine solution. *p*-AV was calculated based on Equation (3):

(3) $$p\text{-AV}=25\times \frac{1.2\times A_{\mathrm{II}}-A_{I}}{w}$$

The TOTOX value was obtained on the basis of Equation (4), showing an overall assessment of the degree of oil sample oxidation [22]:

(4) TOTOX value=p-AV+2×PV

The specific extinctions in UV (K232 and K268) are highly sensitive indices based on the extent to which lipid oxidation developed in oils can be appreciated. Conjugated dienes (CDs) are detected at 232 nm and conjugated trienes (CTs) at 268 nm. The changes in the absorption characteristics were measured with the UV–VIS spectrophotometer Specord 205, Analytik Jena Inc. (Jena, Germany) at specified wavelengths according to the ISO standard methods (3656:2011) [42]. The extinction coefficients, K232 and K268, were determined by measuring the absorbance at 232 and 268 nm of a 1% (*w/v*) solution of SFO oil in isooctane with a path length of 1 cm. Isooctane was used as a blank sample.

### 2.5. ATR-FTIR Spectra Acquisition 

The infrared spectra of untreated and heat-treated oil samples for 4 h and 8 h, respectively, were recorded at room temperature (25 °C) in the range from 400 to 4000 cm^−1^ with a Shimadzu FTIR-8400S spectrometer (Shimadzu Corporation, Kyoto, Japan) equipped with an Attenuated Total Reflectance (ATR) accessory interfaced to a computer operating under Windows based on the Shimadzu IR solution software. The infrared spectra were obtained by subtracting the air reference spectrum. Reference and samples were measured with a scan time of 60 s and a resolution of 4 cm^−1^. A total of 1 mL of the SFO sample was disposed in a thin layer and used to record the FTIR spectra. The spectra were recorded in triplicate as absorbance values at each data point. The fixed-point method was applied to all recorded infrared spectra for baseline corrections [43]. 

### 2.6. Statistical Analysis

All determinations were performed in three replicates and the results were reported as the mean values ± standard deviation (SD). Statistical data processing was performed by one-way analysis of variance (one-way ANOVA). To assess the statistical significance of differences recorded between the mean values obtained for investigated parameters by supplementing SFO with BHT and GPE, the post hoc Tukey test for multiple comparisons and Levene’s test for equal variances were used, respectively. Differences between the analyzed values were considered statistically significant at a probability of *p* < 0.05.

## 3. Results and Discussion

### 3.1. Antioxidant Properties of Grape Pomace and Grape Pomace Extract

The raw grape pomace (RGP), dried grape pomace (DGP) and freeze-dried grape pomace extract (GPE) derived from Pinot Noir grape variety were investigated for antioxidant properties and total phenolic content (TPC) in comparison to the standard antioxidants, ascorbic acid (AA) and butylated hydroxytoluene (BHT).

The antioxidant activity was determined by two different methods, the ferric-reducing antioxidant power (FRAP) assay and 1,1-diphenyl-2-picrylhydrazyl (DPPH) radical scavenging activity. The results on TPC and FRAP values are shown in Table 1.

The data presented in Table 1 show that the residue that remained after the fermentation of Pinot Noir grapes, i.e., raw grape pomace (RGP), consisting mainly of skins and seeds, contains high levels of polyphenols. Many factors impact the TPC in grape marc, such as grape variety, growing region, weather and winemaking techniques [44,45].

Total phenolic content (TPC) in RGP was 71.207 mg GAE/g d.s.—this closely matches the data reported by other authors: Iora et al. [44] (1.2–74.8 mg GAE/g d.s.), Negro et al. [45] (27–53 mg GAE/g d.s.), Rockenbach et al. [46] (30–70 mg GAE/g d.s.).

Consequently, grape pomace, an abundant and renewable wine industry byproduct, can be viewed as a potential source of bioactive compounds for further applications.

A major shortcoming is that this byproduct contains large quantities of water, which has a negative impact on its chemical and microbiological stability. The high moisture content of RGP (52.48%) favors microbial spoilage and enzymatic degradation processes, and consequently affects its subsequent uses. Therefore, an initial conditioning step of RGP is necessary to slow down these processes and ensure a satisfactory shelf life.

Moderate-temperature convective drying is recommended for decreasing the moisture content of plant origin byproducts to values that are not suitable fermentation processes and microbiological alterations [47]. The drying of RGP led to a substantial decrease in water content, from an initial value of 52.48% to a residual content of 4.21% for the DGP sample. In a food system with a moisture below 5%, the water is tightly bound and is not available for biological functions, thus, the spoilage processes through microbial degradation and enzymatic reactions are not supported [48]. The damage of high-value bioactive compounds is limited by moderate-temperature drying, and the bioactive potential of grape pomace is preserved at a high level [47]. As is shown in Table 1, a decrease of approximately 21% in TPC (*p* < 0.05) was recorded in response to raw grape pomace drying, but the dried grape pomace still represents a valuable source of phytochemicals. The only downside of the technique used for RGP conditioning is the long drying time.

In recent years, particular attention has been paid to the recovery of polyphenolic compounds from grape pomace. Ethanol–water mixtures are recommended as an extraction medium for bioactive compounds because ethanol is safe and cheaper than other solvents, such as methanol–water mixtures or acetone, and is preferable for the subsequent use of recovered phytochemicals for food purposes [49]. 

In our study, grape pomace extract (GPE) with a residual moisture content of less than 5% (3.83%) was obtained after the lyophilization of the concentrated extract obtained from DGP.

Studies have focused on the strategies adopted to increase the yield of bioactive phenolics recovered from grape pomace, and have highlighted the crucial role of both the extraction techniques and particular conditions of this process [49,50].

A TPC of 283.514 mg GAE/g d.s. was obtained for the freeze-dried grape pomace extract, in agreement with the data reported in the literature, ranging from 75 to 618 mg GAE/g d.s. [49].

Phenolic compounds are known to contribute to the antioxidant activity of grape pomace and derived extracts, thus, data obtained by the FRAP assay demonstrated high antioxidant activities of investigated samples. The highest value of reduction potential was noticed for AA, followed by BHT, GPE, RGP and DGP. Among RGP, DGP and GPE, the same trend between TPC and its ability to reduce Fe^3+^ ions was observed. The drying process induced significant losses (*p* < 0.05) in FRAP values, but DGP retained 76% of the ferric-reducing antioxidant power of the raw grape pomace. A high antioxidant activity was observed for GPE (2611.291 µM Fe^2+^/g d.s.), but the recorded value was lower than the standard substances, BHT and AA.

**Figure 1:**
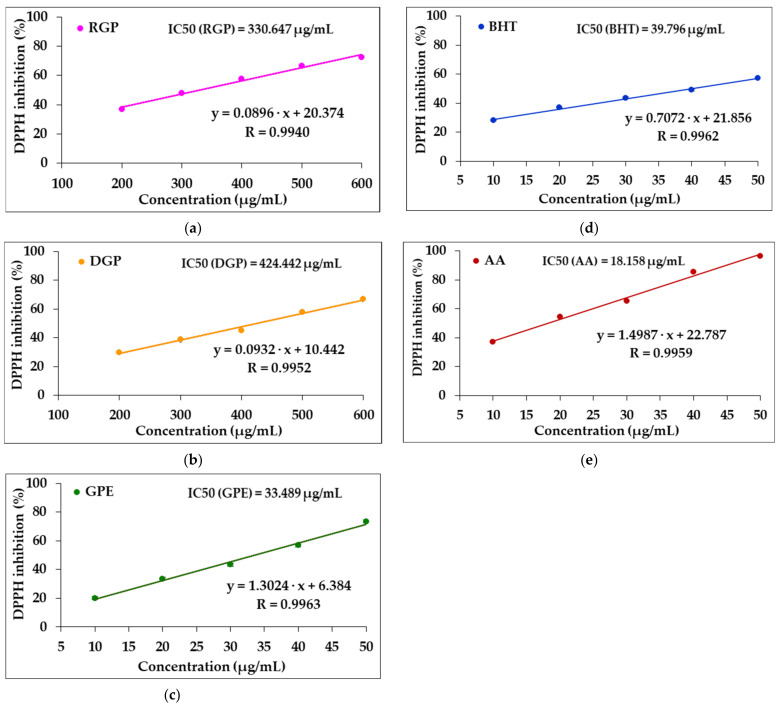
Percentage of DPPH inhibition (%) versus the sample concentration: (**a**) raw grape pomace (RGP); (**b**) dried grape pomace (DGP); (**c**) grape pomace extract (GPE); (**d**) butylated hydroxytoluene (BHT); (**e**) ascorbic acid (AA).

Table 2 reports the DPPH radical inhibition (%) achieved by different concentrations of RGP, DGP and GPE compared to BHT and AA, with these data being used to plot the regression curves of DPPH inhibition (%) against the sample concentration, from which the half-maximal inhibitory concentration (IC50) was estimated, as shown in Figure 1.

It can be seen that for all investigated concentrations, DPPH radical inhibition was higher for RGP compared to DGP. The inhibitory activity towards DPPH radical followed the same pattern as in the case of the total phenolics and FRAP values. The convection drying of grape pomace resulted in some loss of DPPH radical scavenging activity in the range from 7 to 21%, as shown in Table 2.

The free-radical scavenging activity of samples was reported as IC50 (μg/mL), this being the concentration of antioxidant required to achieve a 50% decrease in the initial DPPH concentration. 

IC50 is widely used to provide information on the antioxidant activity of investigated samples—its value is inversely proportional to the sample’s ability to scavenge free radicals [50]. 

The values of IC50 for RGP, DGP and GPE were calculated from the equations of the linear regression curves displayed in Figure 1, compared to those of the standard substances AA and BHT. From these equations in the form y = f (x), where “y” is the DPPH inhibition (%) and “x” is the concentration of the test sample, when the value of “y” (DPPH inhibition, %) is 50, the value of x will be obtained, which becomes the IC50 value.

A lower radical scavenging activity of DGP compared to RGP was registered in response to drying. The corresponding IC50 value increased from 330.647 µg/mL (RGP) to 424.442 µg/mL (DGP), as a direct result of heat exposure in the convection oven. This finding is consistent with the polyphenol content of the samples, so it is possible to attribute the inhibitory activity against DPPH radical to the presence of these bioactive compounds.

Some authors [46,50] noted a close relationship between TPC and antioxidant activity, but others stated that antioxidant activity depends on the phenolic profile [51].

The radical scavenging activity of GPE was lower compared with AA and was comparable with synthetic antioxidant BHT, with IC50 values of 33.489 µg/mL for GPE, 18.158 µg/mL for AA and 39.796 µg/mL for BHT, respectively, as shown in Figure 1.

The results for IC50 in DPPH assay were in the same range as those reported by other authors for grape marc extract, as follows: 25 µg/mL according to Veskoukis et al. [52] and 50 –75 µg/mL found by Saratale et al. [53]. In another study, IC50 values ranging from 88 to 160 µg/mL were reported for grape pomace extract [54]. These data show that the antioxidant activity of grape pomace extracts varied with respect to the antioxidant assays used. The results confirmed that the bioactive compounds present in GPE show both a high ferric-reducing power and free-radical scavenging activity.

### 3.2. Assessing Thermo-Oxidative Degradation of Sunflower Oil Samples by Routine Chemical Indices

#### 3.2.1. Peroxide Value (PV)

Thermo-oxidation of edible oils implies both primary and secondary oxidation. However, secondary oxidation progresses, mainly because of the poor stability of hydroperoxides at elevated temperatures [55]. Hydroperoxides produced in the initial stages of lipid oxidation are chemical compounds with reduced stability and are very sensitive to subsequent reactions which result in the formation of a broad range of secondary oxidation products such as aldehydes, alcohols and ketones. The transient nature of primary oxidation products limits the measurement of hydroperoxides, but they are linked to a potential risk of subsequent formation of undesirable sensory compounds. It is important to mention that a low PV can express both an initial stage and an advanced lipid oxidation process. PV is used as an influential indicator for the primary lipid oxidation process developed in the heat-stressed sunflower oil samples [2].

Data shown in Table 3 provide information on the changes in PV of oil samples with addition of BHT and various concentrations of GPE subjected to heat stress, which are useful to assess the degree of primary oxidation of the oils in relation to the dose used for SFO supplementation.

It can be noted that heat exposure promoted the primary lipid oxidation in sunflower oil leading to significant increases in PV. The peroxide values record increases only when the hydroperoxides formation rate is higher than the decomposition rate [22,56]. In our study, PV increased during heat exposure for 4 h and 8 h, respectively, showing that the rate of hydroperoxides formation was greater than their decomposition rate into secondary oxidation products.

The primary oxidation of lipid, which significantly affects the oxidative status of SFO and consequently alters its quality, was strongly inhibited by supplementing the oil with both 200 ppm of BHT and various concentrations of GPE. 

The statistical analysis of the data by the ANOVA test revealed significant differences in PV between the control sample and the samples with added BHT and GPE after 4 h and 8 h of heat exposure, respectively.

After 4 h and 8 h of thermal treatment, the peroxide value of samples with added BHT decreased by 27% and 23%, respectively, versus the control, while oil samples supplemented with GPE achieved important decreases in peroxide values, ranging from 12% to 47% (after 4 h) and from 14% to 42% versus the control, after 8 h. 

The potential of GPE to limit the oxidative damage by primary oxidation was concentration-dependent. When primary lipid oxidation was monitored over time, a similar inhibitory effect was induced by supplementing the SFO with 200 ppm BHT and 500 ppm GPE.

Our findings on the pattern followed by the PV of oil samples supplemented with natural extracts during heat treatment are consistent with those of other authors who reported a significant delay of lipid oxidation, thus, limiting the oxidative damage depending on the level of supplementing with antioxidants [22,23,56].

#### 3.2.2. The Inhibition of Oil Oxidation (IO)

Apart from the peroxide value, the inhibition of oil oxidation has been considered as a reliable indicator to assess the primary lipid oxidation in SFO subjected to thermal stress [2,22].

**Figure 2:**
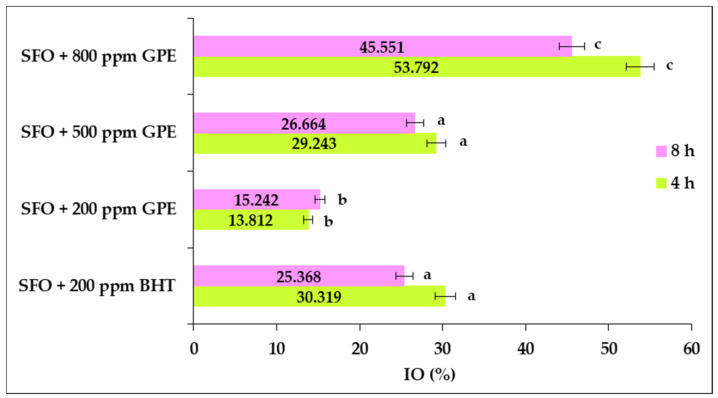
Changes in the inhibition of sunflower oil (SFO) oxidation during convective heat exposure, in response to GPE and BHT supplementation. The values for bars with different letters are significantly different from each other (one-way ANOVA, *p* < 0.05).

The inhibition obtained by the addition of GPE and BHT, on the primary lipid oxidation that occurs in SFO samples after exposure to convective heat, is illustrated in Figure 2. It could be noted that by increasing the level of GPE in oil samples, important increases in IO were noted after both heat exposure periods.

It can be judged that no statistically significant differences (*p* > 0.05) were found for the inhibitory potential on the primary lipid oxidation of BHT at 200 ppm and GPE at 500 ppm, both after the first and second period of heat exposure of oil samples. Therefore, a moderate dose of 500 ppm resulted in an inhibitory effect comparable to that of BHT.

It was also found that a dose of 200 ppm GPE resulted in significantly less inhibition of primary lipid oxidation than BHT, while a concentration of 800 ppm led to a significantly greater inhibition than that attributed to BHT. The results demonstrated that SFO supplemented with a dose of GPE in the range from 200 to 800 ppm showed no pro-oxidative effect during heat exposure up to 8 h.

After the first heating period, the inhibitory action achieved by supplementing the oil with 200 ppm GPE was 54% lower than that of BHT, while a concentration of 800 ppm GPE resulted in a 77% greater inhibitory effect than BHT. At the end of the second heat exposure period, it was found that a dose of 200 ppm GPE resulted in 40% less inhibition that BHT, while a concentration of 800 ppm GPE had an 80% greater inhibitory effect than BHT. 

Statistical analysis revealed that the increase in the GPE dose resulted in significant improvements (*p* < 0.05) of primary oxidation inhibition for both heat exposure periods of oil samples.

#### 3.2.3. *Para*-Anisidine Value (*p*-AV)

For a for more adequate assessment of the evaluation of the lipid oxidation process during oil heating, the quantification of both primary and secondary lipid oxidation products is required. *Para*-anisidine value represents an indicator for the secondary step of lipid oxidation, as it is a measure of the quantity of secondary oxidation products [57]. Data from Table 4 show the changes in *p*-AV in response to the formation of secondary lipid oxidation products during oil heating. These compounds are more stable at high temperatures and include aliphatic aldehydes, ketones, acids, hydrocarbons and alcohols [55,58]. Our data point out that oil heating at an elevated temperature induced a rapid transformation of primary oxidation products to secondary products, of which are responsible for undesirable odors and flavors of vegetable oils. Supplementation of the oil by BHT and GPE led to relevant statistical decreases in *p*-AV (*p* < 0.05) compared to the sample without additives.

After SFO heat exposure for a period of 4 h and 8 h, the *p*-AV of the sample with 200 ppm GPE decreased by 21% and 16%, respectively, versus the control sample. Significant decreases in *p*-AV, relative to the control sample, were also obtained in the range from 14 to 38% after 4 h and from 10 to 33% after 8 h when GPE was used as an additive.

The statistical data analysis by the ANOVA test showed that the extent of secondary lipid oxidation processes in oil samples exposed to convective heat was significantly reduced by the increasing GPE concentration (*p* < 0.05).

GPE addition in sunflower oil did not exhibit pro-oxidative action during heat exposure up to 8 h. This finding is consistent with results that have been reported in other studies on the inhibitory potential achieved by using grape seed extract [22], natural extracts derived from red grape seeds and peels on oxidative processes developed in sunflower oil [23]. As mentioned by other authors [23], the pro-oxidative effect might be due to the increase in the oxidized product content as a result of the long-term exposure to the high-temperature heating of vegetable oils with the addition of additives.

As in the case of primary lipid oxidation, the inhibitory effect of GPE on secondary oxidation processes was concentration-dependent, and closely aligned with the findings of other studies [19,20,22,23]. This statement is explained by the notable antioxidant properties of bioactive compounds found in GPE which are involved in the initiation step of oxidation by the inhibition of free-radical formations; they can also interrupt the propagation of the free-radical chain reaction by working as free-radical scavengers.

The results obtained after convective heat exposure for 4 h and 8 h indicated that no significant differences (*p* > 0.05) were found between SFO samples with BHT and those with the addition of GPE in a concentration of 500 ppm. This finding proves that a dose of 500 ppm GPE exhibited a similar action as BHT with regard to the inhibition of secondary lipid oxidation.

Our results also revealed that GPE at a concentration of 200 ppm was less effective to inhibit lipid oxidation compared to BHT, while GPE applied in a dose of 800 ppm provided more protection against oxidative damage than BHT. The highest concentration of GPE (800 ppm) exhibited the strongest effect to limit the damage that occurred in heat-stressed oil samples as a response to both primary and secondary processes of lipid oxidation.

#### 3.2.4. Total Oxidation Value (TOTOX)

**Figure 3:**
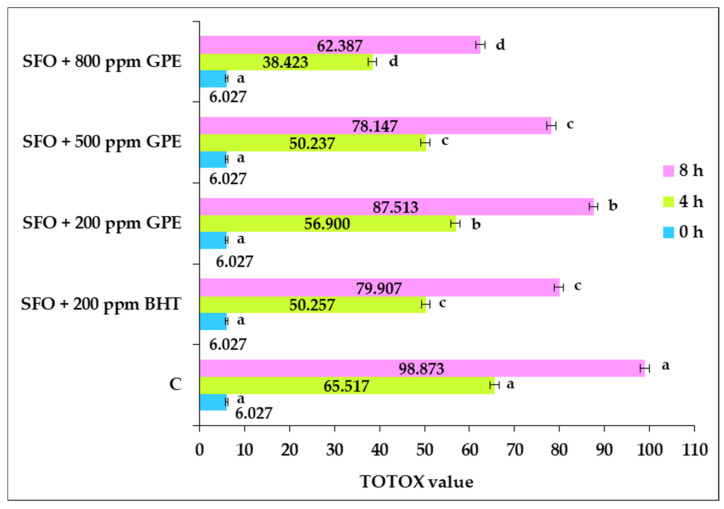
Effect of sunflower oil supplementation with grape pomace extract and BHT on the TOTOX value during exposure to heat. The values for bars with different letters are significantly different from each other (one-way ANOVA, *p* < 0.05).

The TOTOX value is extensively used to estimate the overall oxidative stability of heat-stressed plant oils, and its value can be correlated with the amplitude of oxidative lipid damage [59]. Figure 3 illustrates the changes in TOTOX values associated with the oil samples’ supplementation with BHT and GPE. The TOTOX value provides a global view of the lipid oxidation process developed in the investigated samples.

A significant increase in TOTOX values can be noted as a response to the thermal treatment of oil samples. The most notable increases were recorded in the control sample. The addition of BHT and GPE significantly decreased the TOTOX value versus the control (*p* < 0.05). After 4 h of heat treatment, the addition of GPE to SFO samples induced significant decreases in TOTOX levels, ranging from 13 to 41%, while BHT resulted in 23% of inhibition of lipid oxidation compared to the control sample. Extending the thermal exposure to 8 h resulted in significant decreases in the TOTOX value, with 19% for the oil sample with BHT, ranging from 11% to 37% versus the control sample for the GPE-supplemented samples. The strongest inhibitory effect against lipid oxidation developed during high-temperature heat exposure was observed for the oil samples containing the highest amount of GPE. Thus, for both 4 h and 8 h, the minimum TOTOX values were recorded in the sample containing 800 ppm GPE. The GPE that was applied in a dose of 500 ppm demonstrated an inhibitory action on lipid oxidation, which was similar to that of BHT administered in a dose of 200 ppm. GPE could limit the oxidative deterioration processes occurring in sunflower oil during high-temperature exposure by the fact that the phenolic compounds of natural extract were probably situated at the interface of the lipid system, thus, providing protection against oxidative processes [22].

#### 3.2.5. Specific Extinction Coefficients Measurement

UV absorption changes at 232 and 268 nm, measured as K232 and K268, provide insights on the degree of oxidative damage of lipids [60]. It was found that K232 is strongly correlated with the level of conjugated dienes (CDs) produced as a consequence of the primary oxidation process of polyunsaturated fatty acids, while K268 is more closely linked to the presence of conjugated trienes (CTs) and carbonyl compounds formed in response to secondary lipid oxidation processes [26]. CDs and CTs accumulate and are stable in the oil samples exposed to high temperatures and their assessment has been shown to be adequate to estimate the lipid oxidation status [59]. Changes in K232 and K268 values of the samples supplemented with GPE and BHT during heat exposure are shown in Table 5.

These indices have been used to assess the oxidative deteriorations, and the changes in their values are related to the positional rearrangement of the double bonds of lipid fraction in response to advancing lipid oxidation, and therefore, a part of the non-conjugated system was transformed to conjugated diene and triene double bonds [22,57].

CDs are formed from the primary oxidation of linoleic acid, following the same pattern of peroxides [59], while CTs may be produced both from the primary oxidation of linolenic and dehydration of conjugated diene hydroperoxides [61]. It has been noted that CDs were more effective markers of lipid oxidative deterioration than hydroperoxides [62].

The occurrence of conjugated dienes and trienes in SFO samples at elevated temperatures can be observed by increases in absorbance values at 232 nm and 268 nm, respectively. It was found that the level of CDs registered a continuous increase throughout the investigated heating process. These changes are related to the alterations of lipids due to the conjugation of double bonds as a consequence of primary oxidation.

In addition, data from Table 5 reveal the increases registered in K268, associated with CT accumulation in SFO samples, with the increasing exposure time at a high temperature up to 8 h. These changes are closely related to the generation of secondary lipid oxidation products, usually found in vegetable oils undergoing advanced oxidative processes [26].

The results reported by other researchers reveal that PV, conjugated dienes and trienes of some edible oils such as rapeseed oil, corn oil, soybean oil and sunflower oil gradually increased with the extension of heating. The exposure of vegetable oil to thermal stress led to higher levels of CDs compared to CTs [61].

Oil supplementation with BHT and GPE induces significant decreases in the accumulation of CDs and CTs. It can be said that the antioxidant potential of GPE was highlighted by its ability to reduce the accumulation of CDs and CTs during the heat exposure of GPE-supplemented samples.

After 4 h of heating, the accumulation of CDs was decreased by 25% as an effect of supplementing SFO samples with BHT and by 11–46% versus relative to the control, when GPE was added to SFO at a level ranging from 200 ppm to 800 ppm. In terms of CTs, the addition of GPE led to decreases in the accumulation of conjugated trienes ranging from 11% to 44% versus the control, and of 24% related to the control in the sample with BHT. At the end of convective heat exposure, the addition of BHT reduced the accumulation of CDs by approximately 26% versus the control, while CT’s accumulation was decreased by 20% versus the control. Additionally, supplementation of the SFO with GPE resulted in relevant decreases in CDs and CTs, ranging from 14 to 39% and from 14 to 29%, respectively, compared to the control. The highest level of GPE (800 ppm) exhibited higher ability than BHT to limit the accumulation of CDs and CTs during the oil sample’s exposure to convective heat for both 4 h and 8 h, respectively.

Our results on the efficiency of GPE to improve the thermo-oxidative stability of SFO exposed to high temperature are aligned to those of other studies concerning the significant inhibitory effect on oxidative damage achieved by the addition of natural extracts to sunflower oil exposed to thermal stress [5,57,62]. The strong inhibitory effect demonstrated on lipid oxidation was associated with high-value antioxidant compounds.

### 3.3. Assessing the Thermo-Oxidative Degradation of Sunflower Oil Samples by ATR-FTIR Spectroscopy

Since there is no one standard method for detecting the oxidative alterations of lipids throughout the heat exposure process, a number of different analytical techniques are usually required. 

In this respect, FTIR spectroscopy has been used for the structural characterization of SFO samples to describe the changes induced in their chemical composition as a result of exposure to elevated temperatures. The effect induced by the convective heat exposure of oil samples without additives and those supplemented with BHT and GPE was investigated versus the corresponding untreated samples.

**Figure 4:**
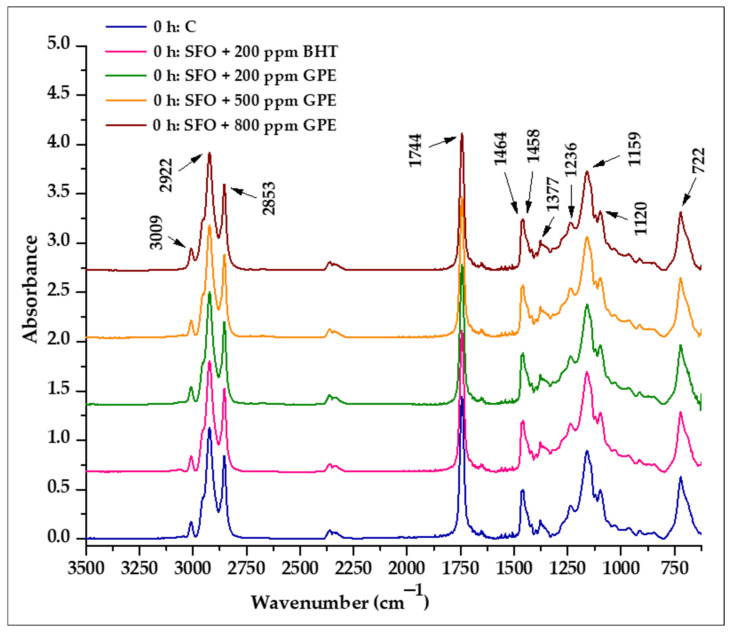
ATR-FTIR spectra of untreated sunflower oil samples supplemented with BHT and GPE compared with the control sample.

As shown in Figure 4, there were no significant differences among the spectra of the untreated oil samples, which means that the band positions and the absorption intensities at the same wavenumber showed high similarity.

**Figure 5:**
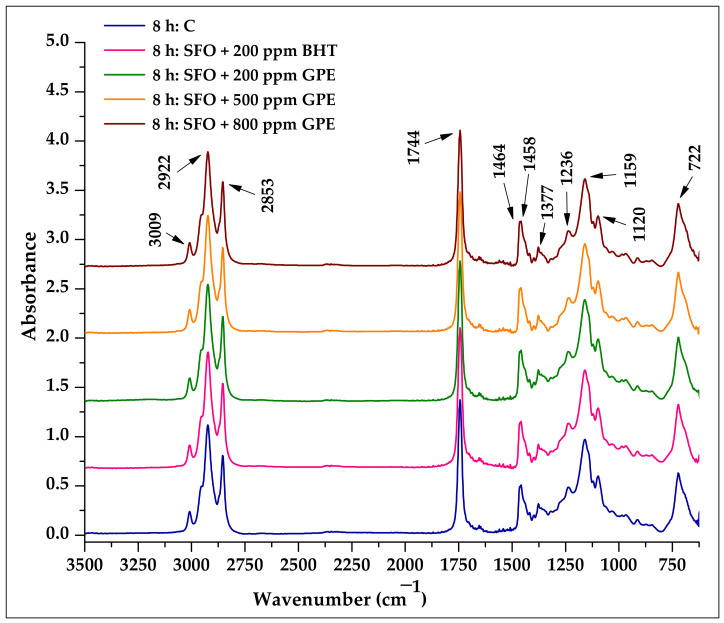
ATR-FTIR spectra of sunflower oil samples supplemented with BHT and GPE compared with the control sample after 8 h of heat exposure.

Figure 5 shows the spectra of the SFO samples after exposure to heat for 8 h. The infrared spectra of the SFO samples exposed to heat for 8 h, at first examination, showed no noticeable differences in spectral characteristics compared to the untreated SFO samples. When the spectra were carefully examined, some changes in the absorption intensity of some specific bands were noticed.

All analyzed oil samples showed absorption bands at the same wave numbers assigned to specific functional groups, as follows: 3009 cm^−1^ (C–H stretching symmetric vibration of the *cis* double bonds HC=CH) [30,32,43,63], strong bands at 2922 cm^−1^ and 2853 cm^−1^ (asymmetric and symmetric stretching vibration of C–H bond in the aliphatic –CH_3_ groups) [30,64,65], the most prominent absorption band at 1744 cm^−1^ (stretching vibration of the ester carbonyl functional groups C=O of the triglycerides) [64,65], 1464 cm^−1^ and 1458 cm^−1^ (bending vibrations of C–H bond in aliphatic –CH_3_ and –CH_2_– groups) [30,32,63,66], 1377 cm^−1^ (symmetric bending vibration of the C–H bond in the –CH_3_ groups) [32,65], 1236 cm^−1^ and 1159 cm^−1^ (stretching and rocking vibration of –C–O ester group, –CH2–) [32], 1120 cm^−1^ (stretching vibration of –C–O ester group) and 722 cm^−1^ (methylene-rocking vibration overlap band –(CH_2_)n– (*n* ≥ 3) with the *cis*-disubstituted olefins out-of-plane vibration) [32,64].

Several researchers have explored the relationship between different absorption ratios and the degree of unsaturation, and their relationship to the thermo-oxidative degradation of fats and oils [31,32,67].

To evaluate the changes that occurred in the degree of unsaturation of oils in response to thermal stress, the following absorbance ratios were calculated [31,32]: RI (A 3009 cm^−1^/A 2922 cm^−1^), RII (A 3009 cm^−1^/A 2853 cm^−1^) and RIII (A 3009 cm^−1^/A 1744 cm^−1^). Additionally, the absorbance ratio RIV (A 1744 cm^−1^/A 2922 cm^−1^) was proposed to investigate the thermo-oxidative degradations [32].

The RI–RIV absorbance ratios calculated from the ATR-FTIR spectral data of untreated and heat-treated oil samples are shown in Table 6.

To eliminate any possible interference of the GPE composition and BHT with the absorbance at the studied wavelengths, the changes in RI–RIV ratios in response to heat exposure were analyzed in relation to the corresponding untreated samples. The subtle discrepancies among FTIR spectra of untreated oils led to small differences in RI–RIV ratios between the control and the samples with BHT and GPE, respectively. These differences could be assigned to the presence of polyphenolic compounds and BHT in SFO samples. In accordance with the literature, the infrared profile of grape pomace extract revealed the absorption bands with peaks around 2850 cm^−1^ and 2920 cm^−1^ assigned to both symmetric and asymmetric stretching vibrations of C–H bonds from (CH_2_) groups, as usually observed for phenolic compounds [68]. Absorption bands around 2956 cm^−1^ and 2872 cm^−1^ due to asymmetric and symmetric stretching vibrations of methyl group are also characteristic of the BHT spectrum [69]. However, the interference of these compounds with absorption at the studied wavelengths is low, as no statistically significant differences (*p* > 0.05) were found in the RI–RIV of untreated samples.

Variations in the investigated absorbance ratios resulted from spectral modifications in the regions 3100–3600 cm^−1^, 2800–3050 cm^−1^ and 1680–1780 cm^−1^ as an effect of the prolonged high-temperature exposure of SFO samples. The noted changes were mainly attributed to primary and secondary lipid oxidation.

The first point that can be noted based on data from Table 6 is that, except for the RIV ratio of the sample with 800 ppm GPE, significant differences in the investigated absorption ratios (*p* < 0.05) were observed between the untreated control samples and all other heat-treated SFO samples, indicating that in response to heat exposure, both changes in the degree of unsaturation and some thermo-oxidative degradation occurred—the changes are more important in the case of prolonged heat treatment.

It was also noted that for the heat-treated control sample for both periods, there were higher values of the RI–RIV ratios, compared to the other heated SFO samples with BHT and GPE, respectively, except for RIII in the 8 h heat-treated control sample, which was significantly lower (*p* < 0.05) than that of the SFO sample, with 800 ppm GPE.

This remark can be justified on the assumption that in an additive-free SFO sample, after both periods of heat exposure, the primary oxidation processes of polyunsaturated fatty acids are more intensive, generating higher quantities of primary oxidation compounds containing *cis* double bonds and conjugate double bonds (as in the case of self-oxidation of oleic acid and linoleic acid) as well as greater amounts of secondary oxidation compounds (aldehydes, ketones, acids, esters) compared to oil samples with BHT and GPE. This observation is in line with those reported by Moharam and Abbas [32] and Van de Voort et al., respectively [70].

For the SFO sample with the addition of BHT, it has been noted that after 4 h of heat exposure, the values of RI–RIV are significantly lower than for the heated control sample. Additionally, slightly lower values of RII and RIII (0.250 and 0.158, respectively) and a significant higher value (*p* < 0.05) of RIV (1.204) were recorded, compared to GPE-supplemented SFO samples, as shown in Table 6. For the RI ratio, no significant changes (*p* > 0.05) were detected between the samples with added BHT and GPE.

After 8 h of heating, the RI–RIII ratios of the SFO sample with BHT are lower than those of the GPE-supplemented samples; on the contrary, the RIV of SFO with BHT showed a higher value than samples with different levels of GPE. The recorded differences are concentration-dependent. The values of the RI–RIV ratios are very close for the SFO samples with 200 ppm GPE and 200 ppm BHT (no significant differences, *p* > 0.05).

The obtained data show that during heat exposure up to 8 h, the SFO supplementation with GPE was more effective in inhibiting thermo-oxidative degradation than BHT—this result is consistent with those reported by Moharam and Abbas [32], Vieira and Regitano-d’Arce [71] and Moreno et al. [72].

By increasing the dose of GPE in oil samples exposed to heat for 4 h and 8 h, increases in RI–RIII ratios and decreases in RIV ratios, respectively, were detected.

It was also noted that after 4 h of thermal treatment, no significant differences (*p* > 0.05) were observed for the RI ratio between SFO samples with different concentrations of GPE. For the RII ratio, the 500 ppm GPE and 800 ppm GPE samples were significantly different (*p* < 0.05) from the 200 ppm GPE sample, while for the RIII and RIV ratios, only the 800 ppm GPE sample was significantly different (*p* < 0.05) from the 200 ppm GPE and 500 ppm GPE samples.

Since after 4 h of heating the RIV ratio decreased with the increasing dose of GPE added to the SFO, it can be said that the amounts of secondary oxidation compounds slightly decrease, thus, limiting thermo-oxidative degradation.

After 8 h of heat exposure, significant differences (*p* < 0.05) were noted between the oil samples with the addition of 200, 500 and 800 ppm GPE in terms of RII and RIV ratios, respectively. Regarding RI and RIII ratios, no statistically significant differences (*p* > 0.05) were observed between oil samples with 200 ppm GPE and 500 ppm GPE, instead, significantly higher changes occurred (*p* < 0.05) when the dose of GPE was increased to 800 ppm.

**Figure 6:**
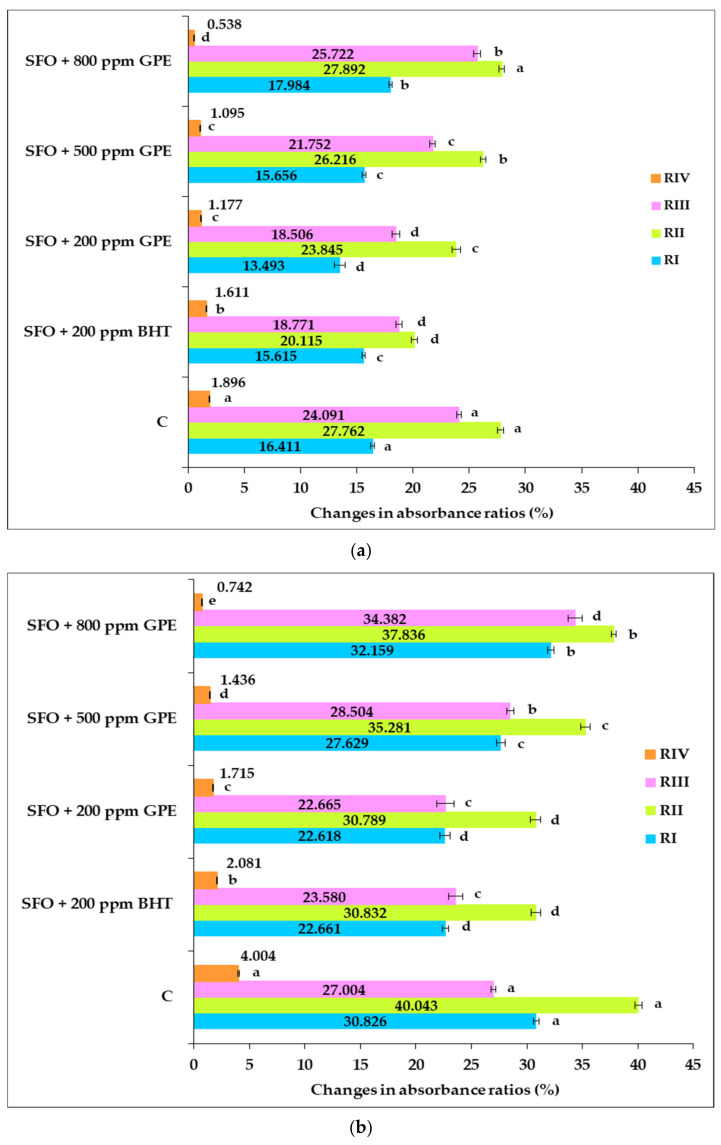
The percentage changes in the absorbance ratios of heat-treated oil samples compared to the corresponding untreated samples: (**a**) after 4 h and (**b**) after 8 h. A 3009 cm^−1^/A 2922 cm^−1^ (RI), A 3009 cm^−1^/A 2853 cm^−1^ (RII), A 3009 cm^−1^/A 1744 cm^−1^ (RIII) and RIV = A 1744 cm^−1^/A 2922 cm^−1^ (RIV). The values for bars with different letters are significantly different from each other (one-way ANOVA, *p* < 0.05).

The data shown in Figure 6 illustrate the percentage changes of the absorbance ratios of heat-treated samples for 4 h and 8 h compared to the corresponding unheated samples.

Based on these data, it can be seen that after 4 h of heat exposure, there were lower growth rates for RII and RIII of the SFO sample with 200 ppm BHT (20.115%; 18.771%) than those of oil samples supplemented with 500 ppm and 800 ppm GPE, respectively, except for RIII of the sample with 200 ppm GPE, where the value was very close to that of the sample with 200 ppm BHT, without statistical significance (*p* > 0.05). This observation is supported by the data from Table 6, revealing that after 4 h of heat exposure, in the SFO sample with added BHT, there is a lower amount of primary oxidation compounds but a higher quantity of secondary oxidation compounds than in the oil samples with added GPE. These results led to the finding that after 4 h of heating, in the sample with the synthetic antioxidant BHT, the primary oxidation proceeded at a lower rate than the secondary oxidation process compared to the GPE-supplemented SFO sample, indicating a more advanced level of thermo-oxidative degradation of the sample with BHT than the GPE-supplemented samples.

Additionally, by prolonging the heating time to 8 h, lower growth rates of RI–RIII ratios were reached for the SFO sample with 200 ppm BHT (22.661%; 30.832%; 23.580%) than those of oil samples with 500 ppm (27.629%; 35.281%; 28.504%) and 800 ppm GPE, respectively (32.159%; 37.836%; 34.382%), except for the sample with 200 ppm GPE, where the values were very close to that of the sample with 200 ppm BHT, with no statistical significance (*p* > 0.05). In terms of RIV, there was a higher rate of increase for the SFO sample with 200 ppm BHT (1.611%; 2.081%) than that of the oil samples with 200 ppm GPE (1.177%; 1.715%), 500 ppm GPE (1.095%, 1.436%) and 800 ppm (0.538%; 0.742%) after 4 h and 8 h of heat exposure, respectively, as shown in Figure 6.

A closer look at the data from Figure 6 reveals that after both periods of exposure to heat, statistically significant differences (*p* < 0.05) were detected in the percentage change of RI–RIV ratios for oil samples boosted with GPE in different concentrations, except for the RIV ratio, when after 4 h of heating, no significant difference was found between the samples with 200 ppm GPE and 500 ppm GPE.

The values increased with the increasing GPE concentration for the RI–RIII ratios, while the RIV ratio value decreased with the increasing dose of GPE used for SFO supplementation.

As an overview, these results may be due to the fact that as the amount of antioxidant compounds provided by GPE in SFO increases, there are more primary oxidation compounds and fewer secondary oxidation compounds after both the first and second period of heat exposure. The rate of decomposition of primary oxidation compounds into secondary oxidation compounds, responsible for thermo-oxidative degradation of oil, has been slowed down by increasing the concentration of antioxidant compounds in SFO. This finding is consistent with the results of other researchers [32,71,72].

All percentage changes for RI–RIV ratios versus the unheated control are greater after 8 h of oil heat exposure than after 4 h. It should also be noted, based on Figure 6, that the percentage changes in RI–RIII ratios for all heat-treated oil samples are considerably higher than that of RIV.

The data obtained reveal the potential of GPE to provide protection against thermo-oxidation—the inhibitory effect obtained by supplementing the oil with a dose of 800 ppm being higher than that of BHT. The results derived from the ATR-FTIR spectra and the investigation of RI–RIV ratios are largely consistent with the findings found in the case of the specific chemical indices analysis of oil samples. This fact proves the efficiency of infrared spectroscopy to be used as a simple research tool to investigate the thermo-oxidative degradation of vegetable oils.

## 4. Conclusions

Our data provide strong evidence for the antioxidant properties of grape pomace extract and its ability to inhibit the degradative thermo-oxidative processes in heat-treated SFO. It is worth noting that Pinot Noir grape pomace is a sustainable source of bioactive compounds and the knowledge of its polyphenolic content and antioxidant properties is useful for other food applications. The investigation of SFO thermo-oxidation by routine indices revealed that GPE applied in a moderate dose of 500 ppm showed a similar effect to BHT against both primary and secondary oxidation, while a level of 800 ppm GPE provided a higher protection against thermo-oxidative degradation than BHT.

The use of FTIR spectroscopy to follow the changes induced in the oil composition as a result of heating at elevated temperatures revealed the suitability of this technique to differentiate untreated and heat-treated samples based on RI–RIV absorbance ratios. Differences found in the absorbance ratios were attributed to primary and secondary lipid oxidation, and their magnitude is impacted by both the type of antioxidant (BHT, GPE) and the dose. This technique also confirms that the potential of GPE to limit thermal oxidative damage was dose-dependent, with the highest level of GPE providing the strongest inhibition. The changes detected in the infrared spectral data are useful indicators of the oxidative stability of edible oils, and the findings revealed by RI–RIV ratios mostly support the results obtained when classical chemical indices were used to monitor the progress of lipid oxidation. For a better characterization of the supplemented oils, further studies will be carried out on the impact of GPE addition at doses of 500 and 800 ppm on the organoleptic properties of SFO. Additionally, the inhibitory effect of GPE will be explored on other edible vegetable oils for comparative studies.

It can be concluded that FTIR spectroscopy stands out as a simple, fast and low-cost technique, able to replace the classic indices used as routine tests for assessing the oxidative stability of vegetable oils. The data reported here allow the promotion of freeze-dried GPE as a potential natural antioxidant to enhance the thermo-oxidative stability of culinary oils during various food applications which involve elevated temperatures.

## Figures and Tables

**Table 1:** FRAP value and total phenolic content (TPC) of the samples tested.

Sample	TPC (mg GAE/g d.s.)	FRAP Value (µM Fe^2+^/g d.s.)
RGP	71.207 ± 1.816 ^a^	637.541 ± 3.461 ^a^
DGP	56.135 ± 1.542 ^b^	482.692 ± 3.047 ^b^
GPE	283.514 ± 2.131 ^c^	2611.291 ± 4.173 ^c^
BHT	-	3421.377 ± 4.306 ^d^
AA	-	4512.131 ± 4.531 ^e^

Raw grape pomace (RGP); dried grape pomace (DGP); grape pomace extract (GPE); butylated hydroxytoluene (BHT); ascorbic acid (AA). Data with different superscript letters reported in the same column are significantly different from each other (one-way ANOVA, *p* < 0.05).

**Table 2:** DPPH radical inhibition (%) achieved by different concentrations of the sample.

Sample	DPPH Radical Inhibition (%)				
	Concentration (μg/mL)				
	200	300	400	500	600
RGP	36.863 ± 0.375	47.769 ± 0.558	57.600 ± 0.574	66.557 ± 0.512	72.260 ± 0.752
DGP	29.863 ± 0.452	38.785 ± 0.542	45.159 ± 0.523	57.953 ± 0.637	66.890 ± 0.711
	10	20	30	40	50
GPE	20.015 ± 0.347	33.520 ± 0.476	43.441 ± 0.591	56.798 ± 0.477	73.502 ± 0.528
BHT	28.056 ± 0.419	37.149 ± 0.548	43.647 ± 0.667	49.011 ± 0.702	57.488 ± 0.724
AA	37.136 ± 0.588	54.347 ± 0.543	65.234 ± 0.655	85.540 ± 0.759	96.479 ± 0.789

Raw grape pomace (RGP); dried grape pomace (DGP); grape pomace extract (GPE); butylated hydroxytoluene (BHT); ascorbic acid (AA).

**Table 3:** Changes in peroxide values (PV) of sunflower oil (SFO) samples with the addition of GPE and BHT during convective heat exposure, compared to the control sample (C).

Heating Time (h)	PV (meq/kg Oil)				
	C	SFO + 200 ppm BHT	SFO + 200 ppm GPE	SFO + 500 ppm GPE	SFO + 800 ppm GPE
0	1.477 ± 0.057 ^a^	1.477 ± 0.057 ^a^	1.477 ± 0.057 ^a^	1.477 ± 0.057 ^a^	1.477 ± 0.057 ^a^
4	12.370 ± 0.501 ^a^	9.047 ± 0.415 ^c^	10.867 ± 0.484 ^b^	9.170 ± 0.442 ^c^	6.507 ± 0.306 ^d^
8	19.753 ± 0.756 ^a^	15.177 ± 0.551 ^c^	16.943 ± 0.586 ^b^	14.867 ± 0.396 ^c^	11.430 ± 0.536 ^d^

Data with different superscript letters reported in the same row are significantly different from each other (one-way ANOVA, *p* < 0.05).

**Table 4:** Changes in *para*-anisidine values (*p*-AV) of sunflower oil (SFO) samples with the addition of GPE and BHT during convective heat exposure compared to the control sample (C).

Heating Time (h)	*p*-AV				
	C	SFO + 200 ppm BHT	SFO + 200 ppm GPE	SFO + 500 ppm GPE	SFO + 800 ppm GPE
0	3.073 ± 0.140 ^a^	3.073 ± 0.140 ^a^	3.073 ± 0.140 ^a^	3.073 ± 0.140 ^a^	3.073 ± 0.140 ^a^
4	40.777 ± 1.948 ^a^	32.163 ± 1.092 ^b^	35.167 ± 1.390 ^b^	31.897 ± 1.408 ^b^	25.410 ± 1.218 ^c^
8	59.367 ± 2.066 ^a^	49.673 ± 0.983 ^c^	53.617 ± 0.929 ^b^	48.413 ± 1.010 ^c^	39.527 ± 1.164 ^d^

Data with different superscript letters reported in the same row are significantly different from each other (one-way ANOVA, *p* < 0.05).

**Table 5:** Changes recorded in K232 and K268 values of sunflower oil (SFO) samples with the addition of GPE and BHT during convective heat exposure, compared to the control sample (C).

**Heating** **Time (h)**	**K232**				
	**C**	**SFO + 200 ppm BHT**	**SFO + 200 ppm GPE**	**SFO + 500 ppm GPE**	**SFO + 800 ppm GPE**
0	0.223 ± 0.008 ^a^	0.223 ± 0.008 ^a^	0.223 ± 0.008 ^a^	0.223 ± 0.008 ^a^	0.223 ± 0.008 ^a^
4	2.011 ± 0.092 ^a^	1.505 ± 0.081 ^c^	1.792 ± 0.072 ^b^	1.424 ± 0.070 ^c^	1.080 ± 0.051 ^d^
8	2.437 ± 0.124 ^a^	1.812 ± 0.102 ^c^	2.104 ± 0.111 ^b^	1.730 ± 0.083 ^c^	1.489 ± 0.084 ^d^
**Heating** **Time (h)**	**K268**				
	**C**	**SFO + 200 ppm BHT**	**SFO + 200 ppm GPE**	**SFO + 500 ppm GPE**	**SFO + 800 ppm GPE**
0	0.071 ± 0.004 ^a^	0.071 ± 0.004 ^a^	0.071 ± 0.004 ^a^	0.071 ± 0.004 ^a^	0.071 ± 0.004 ^a^
4	0.263 ± 0.012 ^a^	0.201 ± 0.008 ^c^	0.235 ± 0.010 ^b^	0.186 ± 0.011 ^c^	0.148 ± 0.007 ^d^
8	0.334 ± 0.015 ^a^	0.267 ± 0.013 ^b^	0.290 ± 0.014 ^b^	0.254 ± 0.013 ^b^	0.237 ± 0.012 ^c^

Data with different superscript letters reported in the same row are significantly different from each other (one-way ANOVA, *p* < 0.05).

**Table 6:** Changes in the absorbance ratios of some specific bands of the infrared spectra of heat-treated oil samples for 4 h and 8 h compared to untreated samples.

Sample	Absorbance Ratio			
	RI	RII	RIII	RIV
	Heating time: 0 h			
C	0.164 ± 0.002 ^a^	0.211 ± 0.002 ^a^	0.137 ± 0.002 ^a^	1.188 ± 0.002 ^a^
SFO + 200 ppm BHT	0.162 ± 0.001 ^a^	0.208 ± 0.001 ^a^	0.133 ± 0.002 ^a^	1.185 ± 0.003 ^a^
SFO + 200 ppm GPE	0.163 ± 0.002 ^a^	0.210 ± 0.002 ^a^	0.135 ± 0.001 ^a^	1.186 ± 0.002 ^a^
SFO + 500 ppm GPE	0.162 ± 0.003 ^a^	0.209 ± 0.003 ^a^	0.133 ± 0.003 ^a^	1.184 ± 0.003 ^a^
SFO + 800 ppm GPE	0.161 ± 0.002 ^a^	0.208 ± 0.003 ^a^	0.132 ± 0.001 ^a^	1.183 ± 0.002 ^a^
	Heating time: 4 h			
C	0.191 ± 0.002 ^b^	0.270 ± 0.004 ^c^	0.170 ± 0.003 ^d^	1.211 ± 0.001 ^d^
SFO + 200 ppm BHT	0.187 ± 0.001 ^b^	0.250 ± 0.003 ^b^	0.158 ± 0.002 ^b^	1.204 ± 0.002 ^c^
SFO + 200 ppm GPE	0.185 ± 0.002 ^b^	0.260 ± 0.005 ^b^	0.160 ± 0.001 ^b^	1.200 ± 0.003 ^b^
SFO + 500 ppm GPE	0.187 ± 0.003 ^b^	0.264 ± 0.004 ^c^	0.163 ± 0.002 ^b^	1.197 ± 0.002 ^b^
SFO + 800 ppm GPE	0.190 ± 0.002 ^b^	0.266 ± 0.002 ^c^	0.167 ± 0.001 ^c^	1.189 ± 0.001 ^a^
	Heating time: 8 h			
C	0.215 ± 0.004 ^d^	0.296 ± 0.004 ^e^	0.174 ± 0.004 ^c^	1.236 ± 0.006 ^d^
SFO + 200 ppm BHT	0.199 ± 0.003 ^b^	0.272 ± 0.005 ^b^	0.164 ± 0.003 ^b^	1.210 ± 0.005 ^c^
SFO + 200 ppm GPE	0.200 ± 0.003 ^b^	0.275 ± 0.003 ^b^	0.166 ± 0.005 ^b^	1.206 ± 0.005 ^c^
SFO + 500 ppm GPE	0.207 ± 0.004 ^b^	0.283 ± 0.003 ^c^	0.172 ± 0.004 ^b^	1.201 ± 0.004 ^b^
SFO + 800 ppm GPE	0.213 ± 0.003 ^c^	0.287 ±0.004 ^d^	0.179 ± 0.005 ^d^	1.191 ± 0.004 ^a^

A 3009 cm^−1^/A 2922 cm^−1^ (RI), A 3009 cm^−1^/A 2853 cm^−1^ (RII), A 3009 cm^−1^/A 1744 cm^−1^ (RIII) and RIV = A 1744 cm^−1^/A 2922 cm^−1^ (RIV). Data with different superscript letters reported in the same column are significantly different from each other (one-way ANOVA, *p* < 0.05).

## Data Availability

The data presented in this study are available on request from the corresponding author.
